# Immunization with individual proteins of the Lrp/AsnC family induces protection against *Brucella melitensis* 16M challenges in mice

**DOI:** 10.3389/fmicb.2015.01193

**Published:** 2015-10-29

**Authors:** Xinhui Wang, Chang An, Mingjuan Yang, Xinran Li, Yuehua Ke, Shuangshuang Lei, Xiaoyang Xu, Jiuxuan Yu, Hang Ren, Xinying Du, Zhoujia Wang, Yefeng Qiu, Bo Liu, Zeliang Chen

**Affiliations:** ^1^Key Laboratory of Zoonosis, Institute of Zoonosis, Ministry of Education, College of Veterinary Medicine, Jilin UniversityChangchun, China; ^2^Experimental Animal Center, Academy of Military Medical SciencesBeijing, China; ^3^Department of Infectious Disease Control, Institute of Disease Control and Prevention, Academy of Military Medical SciencesBeijing, China

**Keywords:** *Brucella melitensis*, protective antigen, Lrp/AsnC transcription regulator

## Abstract

Brucellosis is one of the most common zoonoses worldwide. Subunit vaccines are promising for the prevention of human brucellosis. In our previous protective antigen screening studies, we identified a new protective antigen, BMEI0357, which belongs to the Lrp/asnC protein family, a conserved transcriptional regulator in bacteria that is absent in eukaryotes. In the present study, the *Brucella* genome annotation was screened and a total of six proteins were identified as members of the Lrp/AsnC family. Lrp/AsnC proteins have two domains that are conserved among the family members. However, sequence similarities between these proteins ranged from 9 to 50%, indicating high sequence heterogeneity. To test whether proteins of this family have similar characteristics, all six proteins were cloned and expressed in *Escherichia coli*. The recombinant proteins were purified and their protective efficacy was evaluated in BALB/c mice challenged with *Brucella melitensis* 16M. The results show that all six Lrp/AsnC proteins could induce a protective immune response against *Brucella melitensis* 16M. Antibodies against the Lrp/AsnC proteins were detected in the immunized mice. However, levels of antibodies against these proteins were relatively variable in human brucellosis sera. Taken together, our results show that these six proteins of the Lrp/AsnC family in *Brucella* could induce protective immune responses in mice.

## Introduction

Brucellosis is one of the most common zoonoses worldwide, and is caused by bacteria of the *Brucella* genus. As a zoonosis, brucellosis causes great economic and health losses. The increasing incidence in recent years has shown some characteristics that are different from those observed about 10 years ago (Pappas et al., 2006). New trends in brucellosis make it more challenging: the spread from rural areas to cities, rapid expansion from one place to another, travel-related spread, and long existence in many wild animals (Seleem et al., 2010; Chen et al., 2013).

Brucellosis is a vaccine-preventable disease. Until now, the live attenuated vaccine has been the most efficient vaccine for this infection (Schurig et al., 2002). With extensive vaccination programs, the incidence of brucellosis has decreased to relatively low levels. Indeed, some countries have even announced the complete elimination of brucellosis. However, rigorous inspection programs have shown a resurgence in incidence in many developing countries in the recent years. Live attenuated vaccines have several limitations, including residual virulence, side effects, and reversion to virulent strains. These limitations have restricted their extensive application, particularly for human vaccination.

Subunit vaccines have several advantages over live vaccines that make them appealing for development of new vaccines. A protective antigen is a fundamental requirement of subunit vaccines; therefore, much effort has been made to find ideal antigens for subunit vaccine development (Pasquevich et al., 2009; Perkins et al., 2010). In our previous antigen screening study, we successfully identified a new protective antigen belonging to the Lrp/AsnC protein family that is encoded by the BMEI0357 gene in *Brucella melitensis* (Fu et al., 2012). Proteins of the Lrp/AsnC family are conserved among bacteria but do not exist in eukaryotic organisms (Brinkman et al., 2003). These proteins regulate expression of many other genes within various functional categories (Cho et al., 2008). That is, proteins of this family are involved in regulation of many biological and pathological processes. These characteristics make them good candidates for drug and vaccine development for these pathogens (Peeters and Charlier, 2010).

The Lrp/AsnC protein family may have several members within a particular bacterial genus. Whether *Brucella* also encodes several members of the Lrp/AsnC family remains unknown. We addressed this question and then asked whether these proteins could also serve as protective antigens for use in a brucellosis vaccine. In the present study, we screened the genome annotation of *Brucella* for proteins of Lrp/AsnC family. These Lrp/AsnC family genes were cloned and expressed in *Escherichia coli*, and the protective roles of these recombinant proteins were evaluated by virulent strain challenge *in vivo*.

## Materials and methods

### Mice and ethics statement

Female 6-week-old BALB/c mice were obtained from the Animal Center of Military Medical Sciences. All animals were handled in strict accordance with Experimental Animal Regulation Ordinances defined by the Chinese National Science and Technology Commission. The animal work was approved by the Beijing Institute of Disease Control and Prevention Animal Ethics Committee (BIDCP003-2014). Animals were provided with humane care and healthful conditions during their stay in the facility. All individuals who used animals had received instruction in experimental methods and in the care, maintenance, and handling of mice, and were under the committee's supervision.

### Cloning, expression, and purification of his-tagged recombinant proteins

Genome annotation of *B. melitensis* 16M was screened to find genes encoding proteins of Lrp/AsnC family (DelVecchio et al., 2002). The Lrp/AsnC proteins were cloned and expressed essentially as previously described (Fu et al., 2012). Briefly, the selected open reading frames (ORFs) were amplified by PCR using genomic DNA of *B. melitensis* 16M with specific primers tailed with restriction sites (Table S1). The amplified DNA fragments were digested with appropriate enzymes and subcloned into similarly digested pET28a. The recombinant proteins were expressed in *E. coli* BL21 as N-terminally His-tagged fusion proteins. The expression of the recombinant proteins was verified by SDS-PAGE. The recombinant fusion proteins were then purified by affinity chromatography on Ni^2+^-conjugated chelating sepharose. The purity of the purified proteins was assessed by SDS-PAGE and the concentrations were quantified with a Nanodrop-1000 (NanoDrop, USA).

### Immunization and protection experiments

Female 6-week-old BALB/c mice were immunized via the intraperitoneal (i.p.) route with purified recombinant proteins as described previously (Fu et al., 2012). Briefly, mice were immunized with 200 μL of recombinant proteins (30 μg) or PBS (negative control) mixed with complete Freund's adjuvant (Sigma-Aldrich, St. Louis, MO, USA) on day 0 and with incomplete Freund's adjuvant (Sigma-Aldrich) on day 15. Mice were vaccinated with live attenuated vaccine strain S19 as a positive control. At 30 days after the last immunization, mice were challenged i.p. with 1 × 10^5^ CFU of *B. melitensis* virulent strain 16M. Mice were sacrificed by cervical dislocation 14 days post challenge and spleens were removed aseptically and homogenized with PBS containing 0.1% Triton X-100. The homogenates were serially diluted and plated on TSA plates, and the CFU were counted after 5 days of incubation 37°C.

### Humoral immune response

Sera were collected from the immunized mice at 45 days after the first immunization and immediately prior to the challenges. Antibodies were detected in the sera with ELISA by using the recombinant proteins as coating antigens with standard procedures. ELISA for detecting the antibody responses was performed essentially as previously described (Li et al., 2012). Written informed consent was given to brucellosis patient. Institutional ethics committee approved the use of human sera. Human sera were collected from confirmed brucellosis patients and stored at −20°C before use.

### Statistical analysis

The protective efficacy was expressed as reduction of the mean log_10_ CFU ± SD. The differences between groups were analyzed by ANOVA followed by Tukey's honest significant difference posttest by comparing the groups to one another. For ANOVA, *p*-values of 0.05 were considered statistically significant.

## Results

### Members of the Lrp/AsnC family in *B. melitensis* are conserved in structure but diverse in sequence

**Figure 1:**
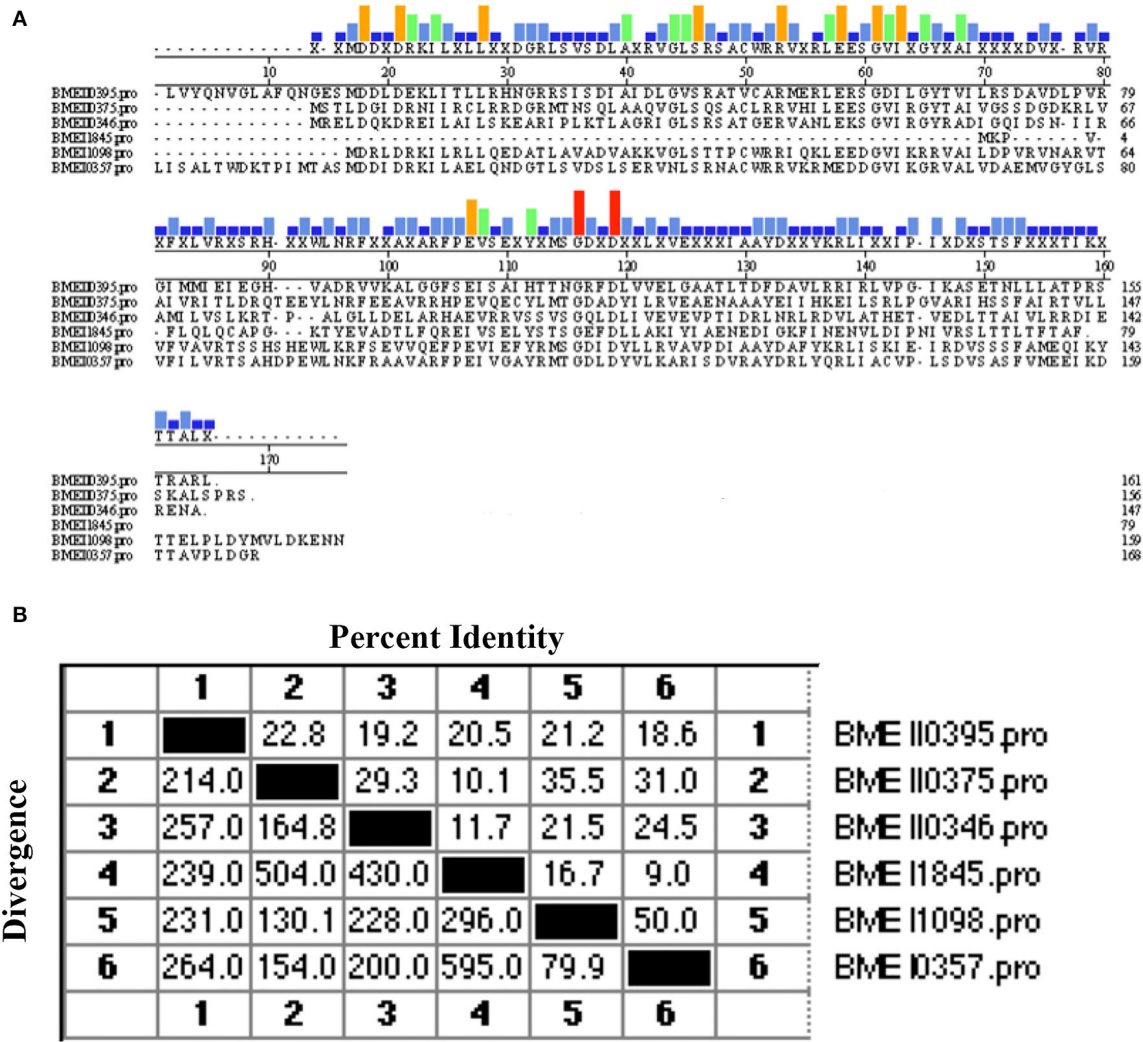
**Sequence similarity between the Lrp/AsnC proteins in *Brucella melitensis*.** Amino acid sequences of the Lrp/AsnC proteins were extracted and aligned with MegAlign. **(A)** Sequence alignment of the six Lrp/AsnC proteins. **(B)** Percent identity of the amino acid sequence of the six Lrp/AsnC proteins.

Of the protective antigens identified in our previous screening study, the antigen encoded by BMEI0357 was demonstrated to induce high levels of cell-mediated immunity and provide protection against virulent strain challenge. Sequence analysis showed that BMEI0357 belongs to the Lrp/AsnC protein family. Careful inspection of the genome annotation showed that there were another five proteins that belong to the Lrp/AsnC family of transcription regulators. As shown in Table 1, five of the six proteins consist of two domains, an N-terminal AsnC-type helix–turn–helix (HTH) domain and a C-terminal AsnC transcriptional regulatory domain. The amino acid length of the five proteins ranged from 146 to 168. One exception to these proteins is BMEI1845, which is 78 amino acids in length and encodes only one AsnC transcriptional regulatory domain. Sequence comparisons between these proteins showed that there were only 9–50% identities among these proteins, implying that sequences of the AsnC protein family are relatively heterogeneous (Figure 1).

**Table 1:** **Domain details of the six proteins of the Lrp/AsnC family^a^**.

**AsnC protein**	**Domain name**	**Accession**	**Description**	**Interval (aa)**
BMEI0357(168aa)	HTH_ARSR	cd00090	Arsenical resistance operon repressor	18–91
	HTH_ASNC	smart00344	Helix_turn_helix ASNC type;	17–125
	HTH_24	pfam13412	Winged helix-turn-helix DNA-binding;	17–64
	AsnC_trans_reg	pfam01037	AsnC family;	83–156
	Lrp	COG1522	Transcriptional regulators	13–165
	PRK11169	PRK11169	Leucine-responsive transcriptional regulator	16–168
BMEI1098 (159aa)	HTH_ARSR	cd00090	Arsenical resistance operon repressor	2–50
	HTH_ASNC	smart00344	Helix_turn_helix ASNC type	1–110
	AsnC_trans_reg	pfam01037	AsnC family;	67–140
	HTH_AsnC-type	pfam13404	AsnC-type helix-turn-helix domain;	1–42
	Lrp	COG1522	Transcriptional regulators [Transcription]	1–149
	PRK11169	PRK11169	Leucine-responsive transcriptional regulator	1–149
BMEI1845 (78aa)	AsnC_trans_reg	pfam01037	AsnC family;	6–75
	Lrp	COG1522	Transcriptional regulators [Transcription]	4–75
BMEII0346 (146)	HTH_ASNC	smart00344	Helix_turn_helix ASNC type	4–107
	HTH_24	pfam13412	Winged helix-turn-helix DNA-binding;	4–51
	Lrp	COG1522	Transcriptional regulators [Transcription]	1–143
	PRK11169	PRK11169	Leucine-responsive transcriptional regulator	2–137
BMEII0375(155aa)	HTH_ARSR	cd00090	Arsenical Resistance Operon Repressor	5–53
	HTH_ASNC	smart00344	Helix_turn_helix ASNC type	4–113
	AsnC_trans_reg	pfam01037	AsnC family;	70–144
	HTH_24	pfam13412	Winged helix-turn-helix DNA-binding;	4–51
	Lrp	COG1522	Transcriptional regulators [Transcription]	1–151
	PRK11169	PRK11169	Leucine-responsive transcriptional regulator	3–133
BMEII0395(160aa)	HTH_ASNC	smart00344	Helix_turn_helix ASNC type	16–120
	HTH_AsnC-type	pfam13404	AsnC-type helix-turn-helix domain;	16–57
	Lrp	COG1522	Transcriptional regulators [Transcription]	11–158
	PRK11169	PRK11169	Leucine-responsive transcriptional regulator	14–156

a *Amino acid sequences of the six Lrp/AsnC proteins were blasted against database with PHI-BLAST. Aligned domain information was extracted and compared*.

### Expression and purification of Lrp/AsnC proteins in soluble form

**Figure 2:**
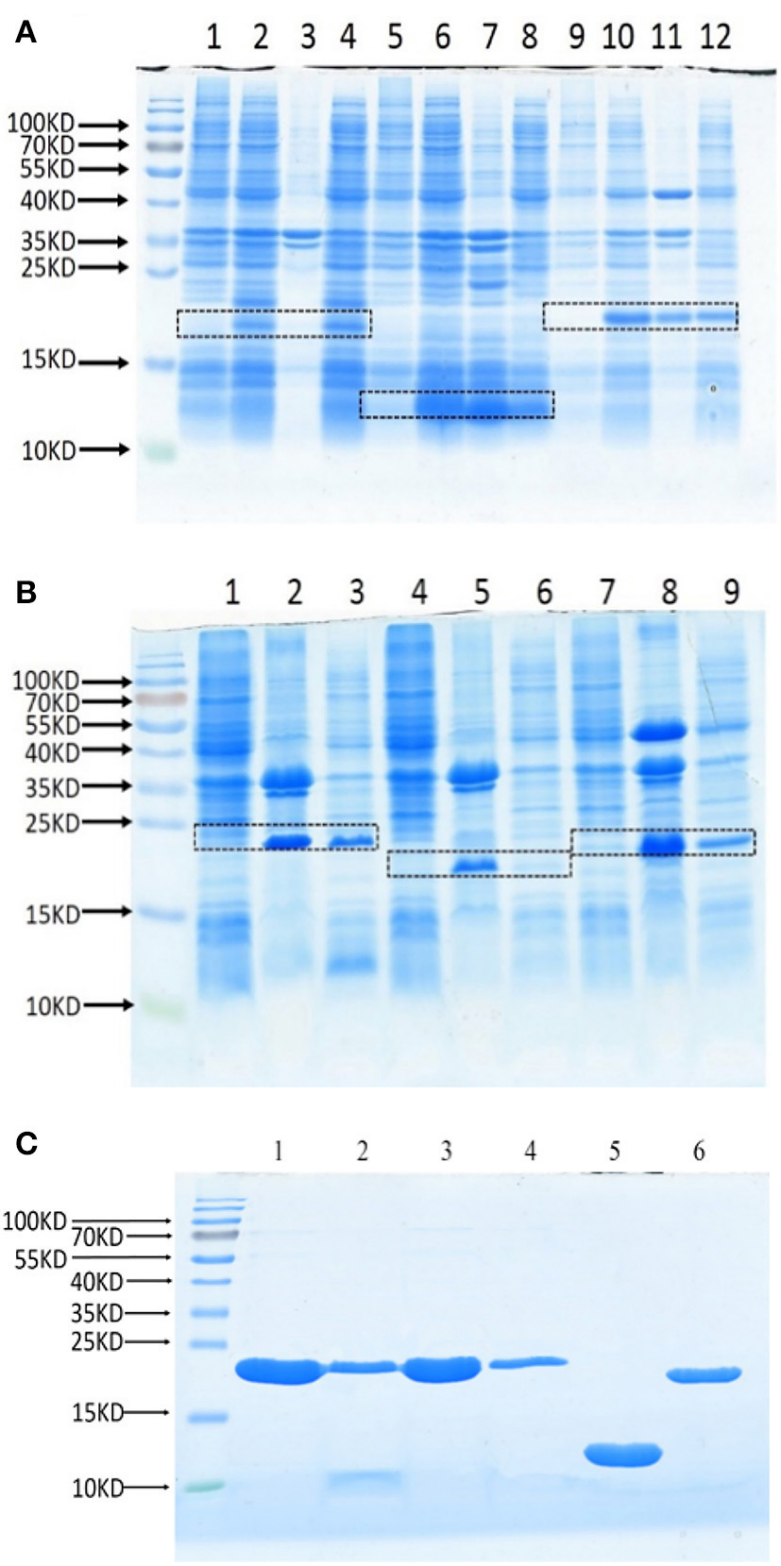
**Expression and purification of recombinant Lrp/AsnC proteins**. Open reading frames of Lrp/AsnC proteins were cloned in expression plasmid pET28a, transformed into *E. coli*, and induced with IPTG. The bacterial lysates were analyzed by SDS-PAGE. **(A)** SDS-PAGE of BMEI1098, BMEI1845, and BMEII0346. Lanes 1, 5, 9: un-induced control; lanes 2, 6, 10: induced lysates; lanes 3, 7, 11: lysate sediments; lanes 4, 8, 12: lysate supernatants. **(B)** SDS-PAGE of BMEII0375, BMEII0395, BMEI0357. Lanes 1, 4, 7: un-induced control; Lanes 2, 5, 8: lysate sediments; Lames 3, 6, 9: lysate supernatants. **(C)** SDS-PAGE of purified proteins. 1–6: BMEI0357, BMEII0395, BMEII0375, BMEII0346, BMEI1845, and BMEI1098.

To test whether these Lrp/AsnC proteins are also protective antigens for *Brucella*, these proteins were cloned and expressed as recombinant proteins. Full-length ORFs were amplified with PCR primers that specifically amplify the coding sequences. The PCR products were digested and then subcloned into expression plasmid pET28a to generate the recombinant expression plasmids. DNA sequencing results showed that all these genes were correctly subcloned. Expression of recombinant proteins was induced with IPTG and tested by SDS-PAGE, the results of which indicate that all these proteins were successfully expressed (Figures 2A,B). Sediments and supernatants of the bacterial lysates were analyzed by SDS-PAGE to test the expression forms. Proteins were expressed in both inclusion bodies and soluble forms. For better activity, recombinant proteins were purified with Ni-NTA from soluble lysates. All six recombinant proteins were successfully obtained with good purity (Figure 2C).

### Immunization with Lrp/AsnC proteins induces protection against 16M challenge in mice

**Figure 3:**
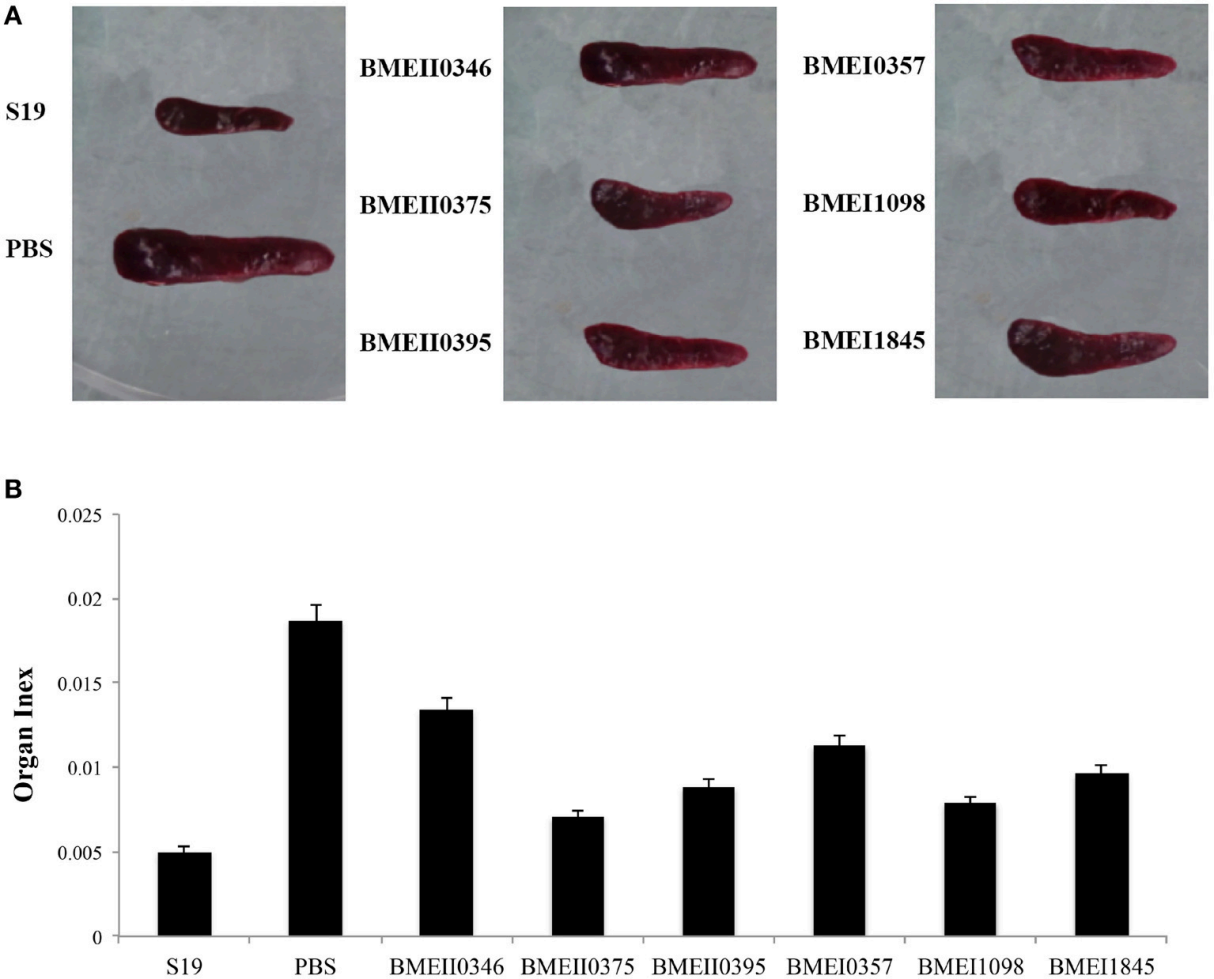
**Reduction of spleen inflammation in the immunized mice after challenge**. Immunized mice were challenged with 16M and 14 days post challenge the spleens were isolated and photographed. **(A)** Immunization reduced the spleen inflammation and organ index **(B)** of the challenged mice.

To evaluate the possible roles of the Lrp/AsnC proteins in induction of protective immune responses, BALB/c mice were immunized with the purified proteins and then challenged with *B. melitensis* virulent strain 16M. For comparison, live attenuated vaccine strain S19 and PBS were used as positive and negative controls, respectively. Groups of six mice were immunized with recombinant proteins, S19, or PBS, and then at 45 days after the first immunization, mice were challenged with 16M. Spleens were isolated from the mice at 14 days post challenge, and bacteria in the spleens were enumerated. As shown in Table 2, compared with the PBS group, the bacterial number in spleens from the S19 group decreased significantly, indicating that immunization of S19 had reduced the bacterial number in the spleen and the protective efficacy of S19 was 1.77 logs. Very interestingly, all six recombinant Lrp/AsnC proteins showed significantly decreased bacterial numbers in spleens compared with the negative control. Of the six proteins, BMEI1098 (1.46 logs) and BMEI1845 (1.46 logs) showed the highest protection levels, followed by BMEII0375 (1.5 logs), BMEII0395 (1.4 logs), BMEII0346 (1.4 logs), and BMEI0357 (1.2 logs). Compared with the other proteins, BMEI0357 appeared to offer the lowest level of protection. To record the reduction in inflammation caused by the virulent strains, isolated spleens were photographed and compared. Compared with non-immunized mice, spleen inflammation and organ index was reduced in the protein-immunized mice (Figure 3). Taken together, these data indicate that immunization with the Lrp/AsnC proteins could induce protection against virulent *Brucella*.

**Table 2:** **Protection efficacies of the Lrp/AsnC family proteins**.

**Vaccine protein**	**Adjuvant**	**Log_10_ cfu/spleen**	**Unit of protection**	***P*-value^a^**
BMEI0357	CFA/IFA	4.83 ± 0.25	1.2	0.0019
BMEI1098	CFA/IFA	4.57 ± 0.25	1.46	0.0009
BMEI1845	CFA/IFA	4.57 ± 0.25	1.46	0.0002
BMEII0346	CFA/IFA	4.63 ± 0.53	1.4	0.0113
BMEII0375	CFA/IFA	4.53 ± 0.58	1.5	0.0119
BMEII0395	CFA/IFA	4.63 ± 0.50	1.4	0.0094
S19	IFA	4.26 ± 0.21	1.77	0.0003
PBS	CFA/IFA	6.03 ± 0.15	–	–

a *Log10 cfu/spleen of immunized group is compared with that of PBS control with t-test, p < 0.05 is statistically significant*.

### Antibodies against Lrp/AsnC proteins detected in immunized mice sera and human patient sera

**Figure 4:**
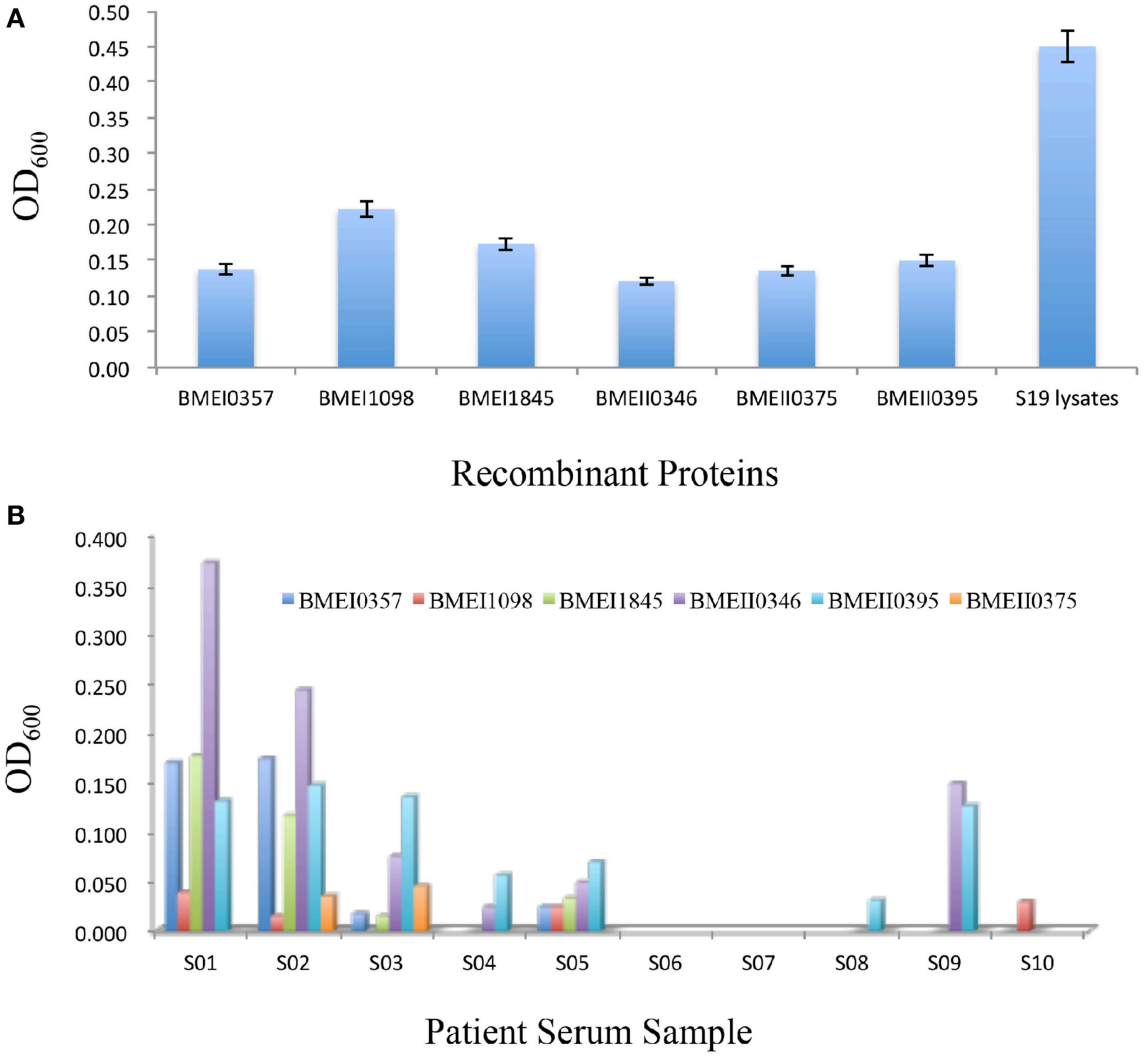
**Antibody responses induced by Lrp/AsnC family proteins**. Purified recombinant proteins were used as coating antigen to detect antibodies in immunized mice sera **(A)** and brucellosis patient sera **(B)**.

Although antibody responses play less important roles in protection against *Brucella* infection, immunization using live attenuated vaccine or antigens also induced antibody responses. To test whether Lrp/AsnC family proteins also induced antibody responses, sera were collected from immunized mice at 45 days after the first immunization. Antibodies were detected with ELISA by using the immunization proteins as coating antigens. As shown in Figure 4A, all these proteins induced high levels of antibodies at 45 days post immunization. Of these proteins, BMEI1098 induced the highest level of antibodies, followed by BMEI1845 and BMEI0395. Antibody level was not significantly correlated with protection unit for these proteins (*p* = 0.5166).

The successful detection of antibodies against Lrp/AsnC family proteins in sera from immunized mice implied that these antibodies might also exist in sera of human brucellosis patients. To test this hypothesis, sera were collected from brucellosis patients and detected by ELISA using the purified recombinant proteins as coating antigens. As shown in Figure 4B, antibodies to one or more of these proteins were detected in 8 of the 10 patient sera. However, antibody titers differed among the sera and proteins. Antibodies to BMEI0395 were detected in 7 of the 10 sera. BMEI0346 reacted with six of the sera samples. For sera samples, S02 reacted with all six proteins, followed by S01, S05, and S03 sera samples. Sera samples S06 and S07 did not react with any of the proteins. These data indicate that although antibodies to Lrp/AsnC proteins exist in patient sera, the distribution differs in terms of both proteins and patient sera.

## Discussion

Bacteria have the capability to adapt to various environments, which is important for their survival and virulence (Celli, 2006). To realize these adaptations, bacteria have evolved many sensing and response systems that regulate gene expression. The Lrp/AsnC family of transcriptional regulators was firstly found to be involved in amino acid metabolism regulation. This regulation system is widely distributed among different bacteria but is absent in eukaryotes (Brinkman et al., 2003; Peeters and Charlier, 2010). Therefore, proteins of this regulatory family are good candidates for vaccines and drug targets for pathogenic bacteria. In our protective antigen screening, we identified a new protective antigen, BMEI0357. Sequence analysis showed that BMEI0357 belongs to the Lrp/AsnC protein family. As demonstrated in other bacterial genera, the Lrp/AsnC family typically has several members. This prompted us to ask whether there are other members of the Lrp/AsnC family and whether these proteins also have protective roles.

Genome sequence analysis revealed a wide phyletic distribution of full-length Lrp-like transcriptional regulators among prokaryotes. In 53% of the analyzed genomes, one or more Lrp-like transcriptional regulators could be identified. About 45% of the bacterial and 94% of the archaeal genomes contain at least one Lrp homolog. In some bacterial genomes, over a dozen of Lrp paralogs are present, whereas in others only a few, or none are observed. Some bacterial endosymbionts without Lrp paralogs completely depend on their host for the supply of amino acids and other key metabolites (Brinkman et al., 2003). The genome annotation of the *B. melitensis* 16M was screened for members of the Lrp/AsnC family, resulting in the identification of six genes. Proteins of the Lrp/AsnC family usually have two domains: an AsnC transcriptional regulatory domain and an Lrp domain. Except for BMEI1845, all these putative Lrp/AsnC proteins contained these two domains (Table 1).

To analyze the potential protective roles of the Lrp/AsnC proteins, ORFs of the six genes were subcloned into the pET28a expression plasmid and expressed in *E. coli*. All six proteins were expressed in both soluble and inclusion-body form. Proteins were purified in their soluble forms from supernatants and used to immunize BALB/c mice. Effective protection was confirmed by challenging the mice with virulent 16M strain. Compared with non-immunized control, bacteria isolated from the spleens of the immunized mice were significantly reduced in number, indicating that immunization with the recombinant proteins had induced protective immune responses. As expected, immunization with BMEI0357 induced protective immune responses. The protective efficacies of the recombinant proteins were slightly lower than that of S19, a live attenuated strain of *Brucella*. This result indicates that immunization with recombinant Lrp/AsnC proteins could provide significant protection. Because the immunizing protein antigens are purified from *E. coli*, there might be some residual LPS contaminated in the antigens that also induce immune responses. Even with this possibility, the protections are still mainly mediated by the immunization protein antigens, for the procedures are the same as what we used in previous study, where only small part of the proteins are found to be protective antigens (Fu et al., 2012).

Proteins of the Lrp/AsnC family have unique structures that are closely related to their functions. LrpA from *Pyrococcus furiosus* is the first protein of the Lrp/AsnC family, the structure of which has been solved (Sedelnikova et al., 2001). LrpA forms an octamer consisting of four dimers. The N-terminal part of the protein is an HTH domain, a fold generally involved in DNA binding (Cui et al., 1995). This N-terminal HTH has been predicted for several Lrp-like proteins. The HTH of LrpA is connected through a hinge to its C-terminal domain (Sedelnikova et al., 2001). In *E. coli* Lrp, the C-terminal domain has been shown to be involved in the response to leucine and activation of transcription. The C-terminus of Lrp-like proteins has a β*αββαβ*-fold or αβ-sandwich, in which the two α-helices are located at one side of the four-stranded antiparallel β-sheet. LrpA forms a homodimer mainly through the interactions between the β-strands of this C-terminal domain, and an octamer through further interactions between the second α-helix and fourth β-strand of the β*αββαβ* motif (de los Rios and Perona, 2007). In the present study, we strikingly found that all the Lrp/AsnC proteins could induce protective immune responses. Because the sequence identities of these proteins were relative low, their protective roles might be primarily determined by their structural characteristics. Previous studies have shown that multimeric proteins are good inducers of immunity. Proteins of the Lrp/AsnC family can form multimers, therefore, it is possible that protective immunity might be induced by the multimeric forms of these proteins. However, this does not exclude other possibilities. Because protection against *Brucella* is mainly mediated by cell-mediated immunity, it is possible that these proteins generate similar T-cell epitopes (He et al., 2010). It will be interesting to investigate into protection mechanisms of Lrp/AsnC proteins.

To characterize the immunogenicity of the recombinant proteins, antibodies were detected in sera samples from the immunized mice. The results show that all the proteins induced antibodies after immunization. In our previous study, BMEI0357 was found to induce cell-mediated immune responses, as demonstrated by the observation that re-stimulation of S19-immunized spleens induced significant titers of IFN-gamma. Since cell-mediated immunity is the main response that provides protection, these proteins are possibly inducers of cell-mediated immunity. In our future work, we will further characterize the cell-mediated immunity of these proteins. Lrp/AsnC proteins form multimers, a unique characteristic of these proteins that makes them good inducers of immune responses. The six proteins differ from each other in terms of amino acid sequence, but all these proteins could induce protective immune responses. At present, it remains to be defined whether this characteristic resulted from the formation of multimers. Therefore, it is of great interest to the test roles of multimers in the protective efficacy of the vaccine.

To test whether humoral responses were induced in human patients, the recombinant proteins were used as coating antigens and their antibodies were detected in the sera obtained from brucellosis patients. Of the 10 sera found to be positive by a plate angulation test, eight were positive for antibodies to the Lrp/AsnC proteins. The two negative sera samples might have resulted from the detection method and antigens. The plate angulation test uses whole-cell antigens, and the reactivity is mediated mainly by LPS from bacteria. Antibody titers for these proteins differed. Taken together, these data suggest that the Lrp/AsnC proteins induced different antibody responses during human infection. The reasons for this low reactivity might include: the Lrp/AsnC proteins were poor inducers of humoral responses, they induced very low levels of antibodies, or they cross-reacted with other proteins and were removed from the analysis.

In summary, the Lrp/AsnC proteins in Brucella were highly heterogeneous in terms of amino acid sequence, but conserved in terms of structural domains. Immunization with recombinant proteins of this family induced protective immune responses against virulent strain challenge in mice. Taken together, our results show that proteins of the Lrp/AsnC family are protective antigens for *Brucella* in mice, and this is the first report to show the protective roles of Lrp/AsnC proteins.

### Conflict of interest statement

The authors declare that the research was conducted in the absence of any commercial or financial relationships that could be construed as a potential conflict of interest.
