# Supplementary material for: Immunization with individual proteins of the Lrp/AsnC family induces protection against Brucella melitensis 16M challenges in mice
Source: Front Microbiol. 2015 Oct 29;6:1193. doi: 10.3389/fmicb.2015.01193 (PMC4625564; doi:10.3389/fmicb.2015.01193)
Supplement: Supplementary file 1 [file Table1.DOCX]

Table S1 Primers used in this study

| Primers | Sequences (5'-3') |
| --- | --- |
| BMEI1098-E-F | AGCTGGATCCATGGACAGGCTCGACAGG |
| BMEI1098-E-R | TCGACTCGAGTGTTGTTTTCTTTGTCGAG |
| BMEI1845-E-F | AGCTGGATCCATGAAACCCGTTTTCCTGC |
| BMEI1845-E-R | TCGACTCGAGTGAACGCGGTGAAAGTCAG |
| BMEII0346-E-F | AGCTGGATCCATGCGGGAACTGGATCAG |
| BMEII0346-E-R | TCGACTCGAGTGGCATTTTCACGCTCAATA |
| BMEII0375-E-F | AGCTGGATCCATGTCTACGCTCGACGGAAT |
| BMEII0375-E-R | TCGACTCGAGTGGAACGCGGCGACAGCGC |
| BMEII0395-E-F | AGCTGGATCCTTGGTTTATCAGAATGTCG |
| BMEII0395-E-R | TCGACTCGAGTTAGCCGGGCGCGGGTTGAAC |
